# Ultrasonography Features of the Plantar Fascia Complex in Patients with Chronic Non-Insertional Achilles Tendinopathy: A Case-Control Study

**DOI:** 10.3390/s19092052

**Published:** 2019-05-02

**Authors:** Carlos Romero-Morales, Pedro Javier Martín-Llantino, César Calvo-Lobo, Daniel López-López, Rubén Sánchez-Gómez, Blanca De-La-Cruz-Torres, David Rodríguez-Sanz

**Affiliations:** 1 Faculty of Sport Sciences, Universidad Europea de Madrid, Villaviciosa de Odón, 28670 Madrid, Spain; carlos.romero@universidadeuropea.es (C.R.-M.); pejamalla@gmail.com (P.J.M.-L.); rusanc02@ucm.es (R.S.-G.); davidrodriguezsanz@ucm.es (D.R.-S.); 2 Faculty of Health Sciences, Institute of Biomedicine (IBIOMED), Universidad de León, 24401 Ponferrada, Spain; ccall@unileon.es; 3 Research, Health and Podiatry Unit, Department of Health Sciences, Faculty of Nursing and Podiatry, Universidade da Coruña, 15403 Ferrol, Spain; 4 Facultad de Enfermería, Fisioterapia y Podología, Universidad Complutense de Madrid, 28040 Madrid, Spain; 5 Department of Physiotherapy, University of Seville, 41009 Seville, Spain; bcruz@us.es

**Keywords:** ultrasonography, Achilles tendon, diagnostic, imaging, tendinopathy

## Abstract

Purpose: The goal of the present study was to assess, by ultrasound imaging (USI), the thickness of the plantar fascia (PF) at the insertion of the calcaneus, mid and forefoot fascial locations, and the calcaneal fat pad (CFP) in patients with Achilles tendinopathy (AT). Methods: An observational case-control study. A total sample of 143 individuals from 18 to 55 years was evaluated by USI in the study. The sample was divided into two groups: A group composed of the chronic non-insertional AT (*n* = 71) and B group comprised by healthy subjects (*n* = 72). The PF thicknesses at insertion on the calcaneus, midfoot, rearfoot and CFP were evaluated by USI. Results: the CFP and PF at the calcaneus thickness showed statistically significant differences (*P* < 0.01) with a decrease for the tendinopathy group with respect to the control group. For the PF midfoot and forefoot thickness, no significant differences (*P* > 0.05) were observed between groups. Conclusion: The thickness of the PF at the insertion and the CPF is reduced in patients with AT measured by USI.

## 1. Introduction

Chronic Achilles tendinopathy (AT) is one of the most common conditions of the ankle and foot, characterized by the combination of pain, swelling and a deficit of functionality in the lower limb [1]. The reported incidence rate was estimated at about 2.35–2.16 per 1000 adults [2]. Recent studies revealed that the presence of disorganization and degeneration in collagen fibers, especially associated with obesity and diabetes disorders, as well as changes in vascularity were risk factors for AT [3]. In addition, changes in tendon thickness and cross-sectional area (CSA) in response to load adaptations were related in patients with AT [4]. Currently, two types of AT were described according to the following locations: Non-insertional (2 to 6 cm from the calcaneus insertion of the tendon) and insertional (at the calcaneus insertion of the tendon).

The Achilles tendon complex may be considered the largest tendon in the body. Its architecture is designed to support tensile loads during its contraction or elongation, and it is susceptible to overuse injuries [5]. In addition, its location makes it a fundamental structure for ankle mobility and locomotion [6]. The Achilles tendon works in a coordinated manner with the gastrocnemius and soleus muscles [7], and the extrinsic and intrinsic foot muscles [8]. The mechanism of AT remains unclear; Galloway et al. [9] reported that changes in the architecture of the tendon and surrounding structures in response to the mechanical load were related in patients with AT. Those adaptations were associated with changes in plantar fascia (PF) morphology, such as the thickness.

The PF is a soft tissue structure across the plantar foot and provides an important role in ankle and foot biomechanics, being a structure capable of working with greater loads by modifying its thickness and stiffness [10]. For example, Huang et al. [11] reported changes in the thickness of the PF in individuals with pes planus, related to mechanical load changes. 

Several authors have studied PF and CPF thickness in different conditions and populations by ultrasound imaging (USI) [12,13,14]. However, there is a lack of evidence about PF thickness in individuals with AT in comparison with healthy subjects.

Ultrasound imaging (USI) has been employed to assess the thickness and CSA of diverse muscles associated with fascial and musculoskeletal conditions. Regarding the lower limb, Lobo et al. [15] showed a reduction in the thickness and CSA of the flexor hallucis brevis and abductor hallucis in subjects with hallux valgus. The peroneus longus muscle CSA was examined by USI and a reduction was observed in the CSA in individuals with ankle sprains [16]. Angin et al. [10] reported a greater CSA and thickness in PF in subjects with pes planus. In addition, Taniguchi et al. [17] showed a reduced vastus medialis thickness in individuals with knee osteoarthritis. Considering other locations, USI has been useful to evaluate the temporalis, sternocleidomastoid and masseter muscles, showing changes in patients with temporomandibular disorders [18]. CSA of the intrinsic hand muscles has been evaluated by Mohseny et al. [19] with USI in subjects with nerve injuries. In the cervical region, the morphology of the deep cervical muscles were observed by USI while they developed an exercise program in subjects with neck pain [20]. Considering the trunk region, Whittaker et al. [21] related the architecture of the abdominal wall muscles with lumbopelvic pain. Several authors argued that USI is a non-invasive, safe and valid method to examine soft tissues and musculoskeletal disorders [22,23].

For the Achilles tendon complex, prior studies were focused on the tendon thickness and CSA in subjects with AT [13]. However, limited evidence of the surrounding soft tissues has been observed, such as PF and calcaneal fat pad (CFP) by USI [24]. 

The goal of the present study was to assess, using USI, the thickness of the PF at the insertion of the calcaneus, mid and forefoot fascial locations, and the CFP in patients with AT. We hypothesized that these selected soft tissue structures would demonstrate changes in individuals with AT.

## 2. Methods

### 2.1. Design

An observational study was carried out following the Strengthening the Reporting of Observational Studies in Epidemiology (STROBE) [25] guidelines from January to December 2017.

### 2.2. Participants

A total sample of 143 individuals from 18 to 55 years was involved in the study. The sample was divided in two groups: A group composed of the chronic non-insertional AT (*n* = 71) and B group comprised of healthy subjects (*n* = 72). For the AT group, subjects were included if they showed the following features: pain in the mid-portion of the Achilles tendon measuring at least 3 out of 10 points in the pain visual analogue scale (VAS), a decrease in the function and tenderness in the body of the tendon for at least 3 months, and undergoing no physical therapy, analgesic or corticosteroid interventions during the study. Exclusion criteria were as follows: subjects with any systemic disease or infection [26], fracture [27] surgeries, plantar orthoses and lower limb disturbances within the last year [28].

### 2.3. Calculation of the Sample Size

G*Power software was employed for the sample size calculation in order to measure the difference between the chronic non-insertional AT group and control group using the CFP thickness (mm) variable of a pilot study (*n* = 20) that was divided into two groups (mean ± SD): 10 subjects with chronic non-insertional AT (7.27 ± 1.97), and 10 subjects for the healthy group (8.51 ± 0.51). For the sample size calculation, a power of 0.80, an α error of 0.05 and an effect size of 0.86 with a one-tailed hypothesis were employed. In conclusion, a sample of 36 was calculated. Nevertheless, we could include a sample of 143 individuals.

### 2.4. Ethical Statement

The study was approved by the La Princesa Hospital ethics committee (Madrid, Spain). All the subjects included in the study signed the informed consent form. The research is in accordance with the Declaration of Helsinki for human experimentation (Project identification code: 2828A). 

### 2.5. Ultrasonography Measurements

**Figure 1:**
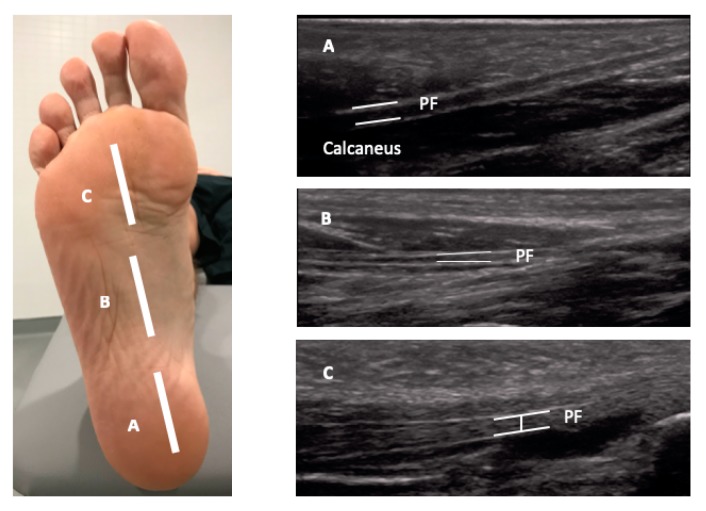
Ultrasound imaging thickness for the plantar fascia at the insertion, midfoot and forefoot locations in an individual with Achilles tendinopathy (AT). Abbreviations: PF, plantar fascia.

**Figure 2:**
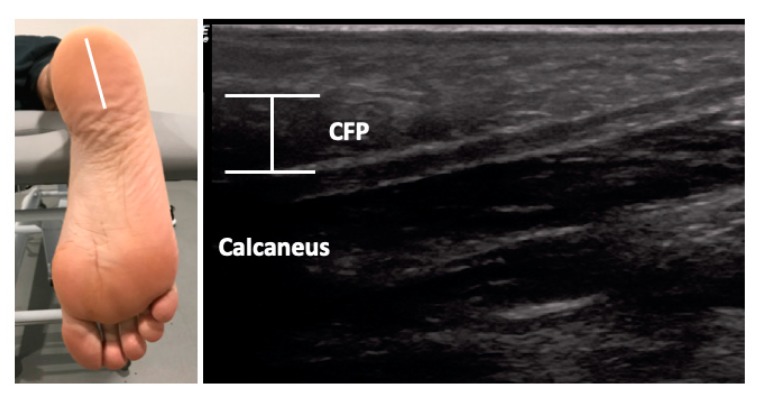
Ultrasound imaging thickness for the calcaneal fat pad in an individual with AT. Abbreviations: CFP, calcaneal fat pad.

The USI assessments were carried out using a high-quality system LogiQ P7 (GE Healthcare; UK) with a 4 to 13 MHz linear transducer (L6-12 RS type, 38 mm footprint). All ultrasound evaluations of the PF were carried out in a supine position with the transducer placed in direct contact with the skin. The PF was scanned in a longitudinal view in all locations. Firstly, for the enthesis at the calcaneus location, the transducer was placed on the line between the medial calcaneal tubercle and the second toe over the PF (Figure 1, A). Secondly, for the midfoot PF assessment, the transducer was located on the same scanning line at the navicular tubercle (Figure 1, B). Thirdly, for the metatarsal region of the PF, the transducer was located on the same longitudinal line near to the second metatarsal head (Figure 1, C) [29]. For the ultrasound examination of the CFP, the subjects were placed in a prone position and the transducer was placed longitudinally at the midpoint of the heel (Figure 2) [24]. According to López et al. [30], the evaluator both flexed and extended the big toe while palpating the subject´s PF to allow accurate identification of the calcaneal tuberosity. All the examinations were carried out by a physiotherapist (P.M.L.) with more than 5 years of experience in ultrasonography. The final scores were collected by the mean of 3 repeated values for each measurement with the ImageJ software (Bethesda, MD, USA).

### 2.6. Data Analysis

Statistical analysis was performed using the SPSS software for Windows (version 22, IBM Corp., Armonk, NY, USA). Firstly, the Kolmogorov–Smirnov test was utilized in order to assess normality distribution. Secondly, the descriptive analysis for the total sample was performed. Finally, a comparative analysis for both groups was developed. The mean, standard deviation (SD) and Student´s *t*-test for independent samples were employed for the parametric data. For the non-parametric data, median, interquartile range (IR) and Mann–Whitney *U* test were realized. Moreover, Levene´s test was performed to assess the equality of variances. The Fisher exact test was used to compare differences between sex and group. In addition, bar graphs completed with the 95 confidence interval (CI) error bars were added in order to illustrate the differences between both groups. For all statistical tests, an α error of 0.05 (95% CI) and a desired power of 80% (β error of 0.2) were employed. 

In addition, a multivariate analysis was carried out using a linear regression (stepwise selection method; *P_in_* = 0.05; *P_out_* = 0.10) in order to predict the influence of the descriptive data and group (presence of Achilles tendinopathy) on the statistically significant outcome measurements (showed in the prior described analyses). The dependent variables were CFP thickness and PF thickness at calcaneus insertion. The independent variables were group, sex, weight, height, BMI and age.

## 3. Results

**Figure 3:**
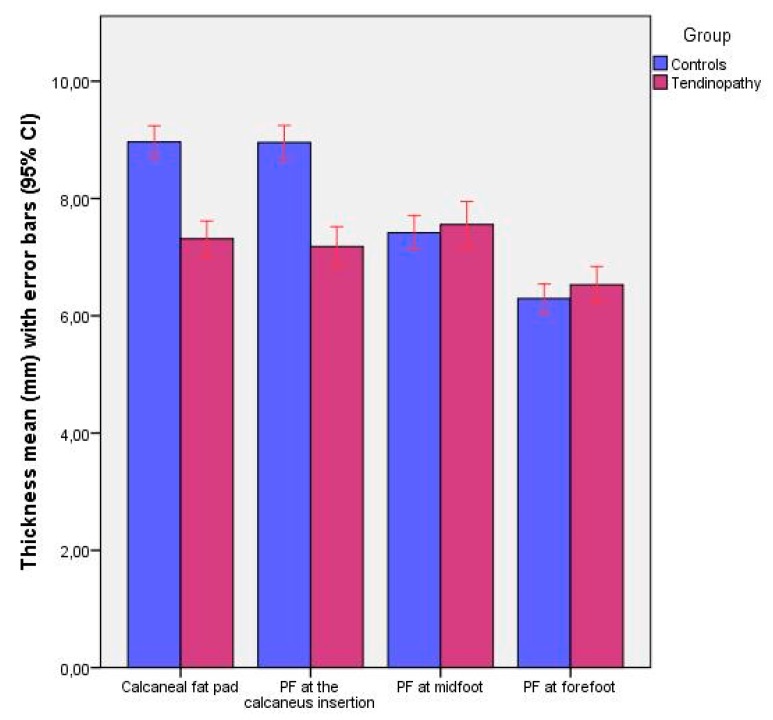
Bar graphs completed with the 95% CI to illustrate the thickness differences of calcaneus fat pad and plantar fascia at the calcaneus insertion, midfoot and forefoot between patients with chronic non-insertional Achilles tendinopathy and controls.

The sociodemographic data did not show statistically significant differences (*P* > 0.05) for the sex, age, weight and height between groups but it did show statistically significant differences (*P* < 0.05) for the body mass index (BMI) between groups (Table 1). Regarding Table 2 and Figure 3, ultrasound evaluations of the CFP and PF at the calcaneus thickness showed statistically significant differences (*P* < 0.01) with a decrease observed for the tendinopathy group with respect to the control group. For the PF midfoot and forefoot thickness, no significant differences (*P* > 0.05) were observed between groups.

According to the linear regression analysis (Table 3), the prediction model for CFP thickness (*R*^2^ = 0.382) was determined by group (presence of Achilles tendinopathy) and sex, and also the prediction model for PF thickness at calcaneus insertion (*R*^2^ = 0.323) was determined by group and weight. The rest of the independent variables did not predict these statistically significant differences between the case and control groups.

## 4. Discussion

USI was considered a valid and reliable imaging method to assess soft tissue structures, architectures and sizes. Considering the strong relationship between the Achilles tendon and PF by their locations and insertions, this study may provide a new approach to the assessment and treatment of individuals with AT. This is the first study where the thickness of the PF and CPF were evaluated with USI in patients with AT, variables of interest due to ankle and foot biomechanics [13].

Foot overpronation was considered a risk factor to the predisposition of AT [31] through disturbances in ankle biomechanics producing an extra mechanical stress on the soft tissue structures, such as the PF. Those findings were related to a thickness decrease in the PF at the calcaneus, a structure that serves as a link between the PF and the Achilles tendon. In addition, Cornwall et al. [32] argued that an excessive foot pronation may increase the foot mobility and the level of stress applied through PF. Many studies have also reported an altered foot arch in patients with disturbances in PF modifies the capability of absorbing ground reaction forces [32,33]. These structural alterations were related to the Achilles tendon capacity to store and release energy during the gait [34]. Our findings showed an altered thickness in the PF at the calcaneus insertion, which could be explained by the changes in the ankle biomechanics, the extrinsic and intrinsic foot muscles observed in patients with AT. 

CFP protects the rear-foot from the stress produced during the initial phase of locomotion and the heel-strike actions. CFP thickness was described as a valid and reliable method to quantify and assess the heel fat pad by USI [24]. Several authors argued that CFP has been implicated in foot and plantar disturbances, such as diabetes [35], fractures [36] and plantar heel pain [37]. To our knowledge, this is the first study to evaluate the CPF thickness in patients with AT. The results of our study showed a decrease in CPF thickness in the tendinopathy group, so it could be considered a relevant variable for the diagnosis and management of individuals with AT.

The findings of the present study did not intend to provide an explanation about the etiology of AT. In addition, several studies argued that the etiology of the tendinopathy was caused by multiple factors. The authors try to offer a novel approach to evaluate and quantify soft tissue structures that usually present disturbances in AT by USI. Thus, USI assessment of the reduction in PF in subjects with AT could be useful to carry out a follow-up for the interventions prescribed to treat AT. 

## 5. Limitations

This study presented several limitations. Firstly, ultrasound M-mode was not used, which could have been convenient for the evaluation of muscle and tissue features. Secondly, a pressure platform was not employed which may have been useful to contemplate load variables, such as plantar pressures.

## 6. Conclusions

The thickness of the PF at the insertion and the CPF is reduced in patients with AT measured by USI. The present study did not intend to provide an explanation of the cause or management of AT. Consequently, USI abnormalities in the PF complex should be interpreted within the clinical context in patients with AT.

## Figures and Tables

**Table 1:** Sociodemographic features, pain scores and VISA-A scale of the sample.

Data	Achilles Tendinopathy (*n* = 71)	Controls (*n* = 70)	*P*-Value Cases vs. Controls
Weight, kg	76.00 ± 12.00 ^†^	75.00 ± 18.50 ^†^	0.412 ^‡^
Age, year	45.11 ± 12.75 *	37.61 ± 11.91 *	0.200 **
Height, m	1.76 ± 0.11 ^†^	1.76 ± 0.12 ^†^	0.566 ^‡^
BMI, kg/m^2^	24.81 ± 2.13 ^†^	23.88 ± 3.67 ^†^	0.012 ^‡^
VAS	2.00 ± 3.00 ^†^	N/A	N/A
VISA-A	56.00 ± 14.00 ^†^	N/A	N/A
Sex, men/women	62/9	54/18	0.086 ^‡‡^

Abbreviations: VAS, visual analogue scale. * Mean ± standard deviation (SD) was applied. ** Student´s *t*-test for independent samples was performed. ^†^ Median ± interquartile range (IR) was used. ^‡^ Mann–Whitney *U* test was utilized. ^‡‡^ Fisher exact test was used.

**Table 2:** Ultrasonography measurements.

Measurement	Tendinopathy (*n* = 71)	Controls (*n* = 72)	*P*-Value
Distance			
Calcaneal fat pad	7.21 ± 1.59 (4.77–11.21) ^†^	8.87 ± 1.59 (6.68–12.74) ^†^	0.000 ^‡^
PF at the calcaneus insertion thickness	6.99 ± 1.84 (4.30–10.93) ^†^	8.94 ± 1.65 (6.11–12.62) ^†^	0.000 ^‡^
PF at midfoot thickness	7.55 ± 1.66 (4.72–12.45) *	7.41 ± 1.24 (4.85–10.43) *	0.579 **
PF at forefoot thickness	6.19 ± 1.55 (4.20–10.84) ^†^	6.32 ± 1.64 (3.83–8.55) ^†^	0.607 ^‡^

Abbreviations: PF, plantar fascia. * Mean ± standard deviation (SD) (minimum–maximum) was applied. ** Student´s *t*-test for independent samples was performed. ^†^ Median ± interquartile range (IR) (minimum–maximum) was used. ^‡^ Mann–Whitney *U* test was utilized.

**Table 3:** Multivariate predictive analysis for CFP and PF thicknesses for patients with Achilles tendinopathy and controls.

Parameter	Model	*R*^2^ Change	Model *R*^2^
CFP thickness (mm)	8.226		
	−1.774 * Group	0.315 ^‡^	
	+0.985 * Sex	0.067 ^‡^	0.382
PF thickness at calcaneus insertion (mm)	7.371		
	−1.818 * Group	0.309 ^‡^	
	+ 0.021 * Weight (kg)	0.023 ^†^	0.323

Abbreviations: CFP, calcaneus fat pad; PF, plantar fascia. * Multiplay: Group (control = 0; Tendinopathy = 1); Sex (women = 0; men = 1). ^†^ *P*-value < 0.05 for a 95% confidence interval was shown. ^‡^ *P*-value < 0.001 for a 95% confidence interval was shown.
